# Myopericytoma occurrence in the liver and stomach space: imaging performance

**DOI:** 10.1186/s12885-017-3146-3

**Published:** 2017-02-20

**Authors:** Zhihua Chen, Wenjie Liang

**Affiliations:** 1 Department of General Surgery, the First people’s Hospital of Taicang City, Taicang Affiliated Hospital of Soochow University, No. 58, Taicang, Suzhou 215400 China; 2 Department of Radiology, the First Affiliated Hospital, College of Medicine, Zhejiang University, 79# Qingchun Road, Hangzhou City, Zhejiang Province 310003 China

**Keywords:** Myopericytoma, Imaging, CT, MRI

## Abstract

**Background:**

Myopericytoma is a rare and usually benign tumor, which is even rarer if it occurs in the liver and stomach space. Previous reports of myopericytoma were mostly related to its pathological manifestations, while imaging reports were rare. Here, we report the computed tomography (CT), computed tomography angiography (CTA) and magnetic resonance imaging (MRI) performance for one deep myopericytoma.

**Case presentation:**

In this study, one deep myopericytoma in the liver and stomach space is reported. A CT that was not contrast-enhanced showed a lobulated tumor with heterogeneous density, and a contrast-enhanced CT showed that the mass had progressive enhancement. CTA showed that the blood-supply of the tumor was supplied by the anterior superior pancreaticoduodenal artery and the left gastric artery. An MRI showed the lesion had isointensity on T1-weighted imaging (T1WI) and slight hyperintensity on T2-weighted imaging (T2WI). The lesion MRI enhancement characteristics were similar to the characteristics from the contrast-enhanced CT. In this case, the enhancement pattern of the tumor was the centrifugal enhancement for both the contrast-enhanced CT and MRI. After surgical resection of the tumor, the pathological diagnosis was myopericytoma, and there was no recurrence in a short-term follow-up.

**Conclusion:**

The myopericytoma generally has a rich blood supply. When there is necrosis in the center lesion, the lesion has peripheral enhancement. Abdominal myopericytoma could be categorized as having centrifugal enhancement.

## Background

Myopericytoma is a rare soft tissue tumor, and the concept of a myopericyte was first proposed by Dictor in 1992 [1]. In 1998, Granter adopted the name myopericytoma for this form of tumor [2]. Afterwards, WHO (2002) named this tumor type myopericytoma in their classification of soft tissue tumors, belonging to the group of peripheral blood cell/vascular cell tumors [3]. It commonly occurs in men, where the incidence is approximately 2 times that in women; the average age of onset is the fifth decade [4]. The four limbs are the most commonly affected areas, and occasionally the intracranial space, nose, periungual, and urinary tract are involved [4–8]. In the past, the reports of these cases were mainly related to clinical pathology and were rare in medical imaging findings. Myopericytoma is generally benign, but when it occurs in a deep location, it is more likely to be a malignant tumor [4]. Therefore, it is necessary for clinicians to understand the imaging findings for these tumors. In this study, we report one case of myopericytoma that occurred in the liver and stomach space and describe the imaging findings in detail, including the computed tomography (CT), computed tomography angiography (CTA) and magnetic resonance imaging (MRI) manifestations. Additionally, we will review the previous literature and summarize the imaging features of these tumors.

## Case presentation

**Fig. 1:**
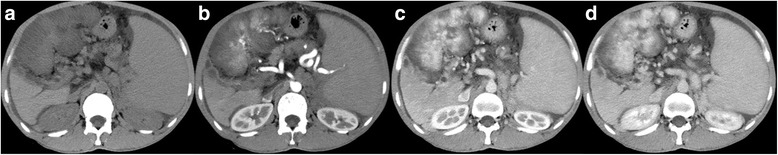
Abdominal conventional and contrast-enhanced CT scan showed a lobulated mass with uneven density (**a**). Arterial phase (**b**) showed partial enhancement of the lesion center, and the venous phase (**c**) and delayed phase (**d**) expanded the enhanced scope, and showed centrifugal enhancement

**Fig. 2:**
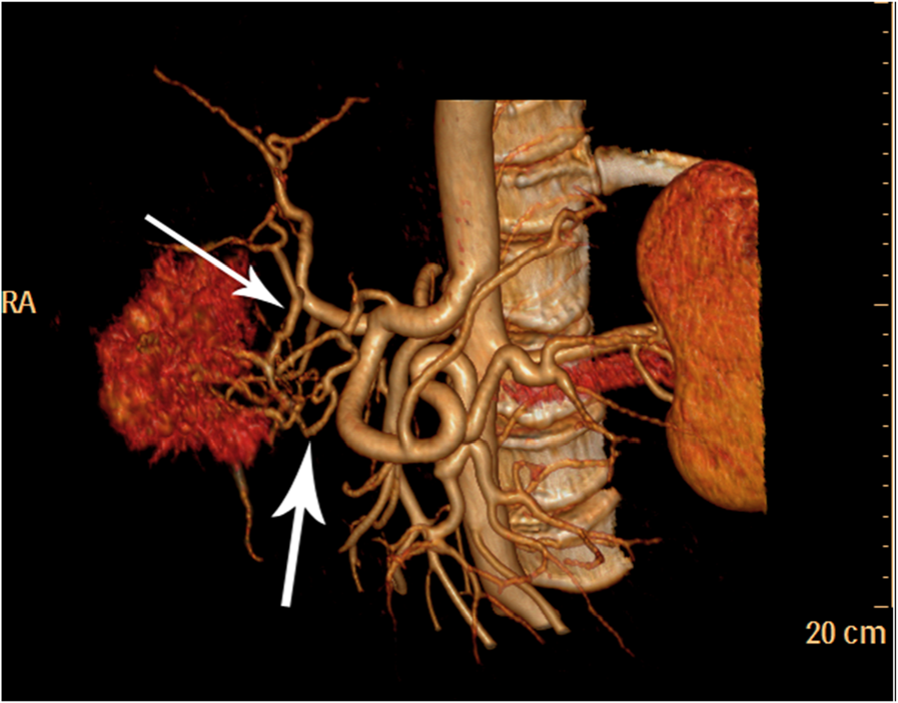
A computed tomography angiography scan showed the anterior superior pancreaticoduodenal artery (*the thin arrow*) and the left gastric artery (*thick arrow*) branches extended to the tumor center

**Fig. 3:**
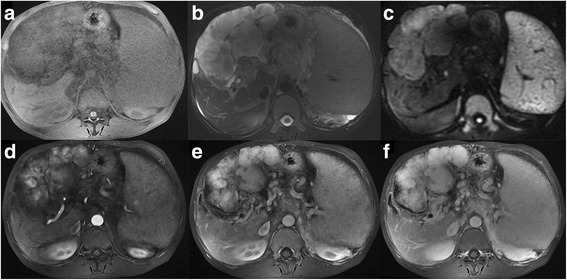
Abdominal conventional and contrast-enhanced MRI scans displayed a soft tissue mass and T1WI was isointense, compared to the muscle signal (**a**). Transverse T2WI showed slight hyperintensity (**b**). The DWI sequence showed slightly hyperintensity (**c**). Transverse artery phase showed the central region enhanced (**d**). In the venous phase (**e**) and delayed phase (**f**), the enhanced range increased, showing centrifugal enhancement

**Fig. 4:**
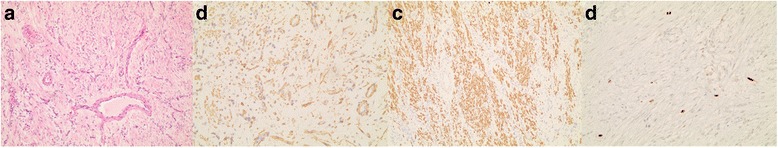
Histopathological examination (H&E, 100×) showed spindle cells arranged in bundles or woven around the vessels (**a**). Immunohistochemistry, SMA (**b**) and Desmin (**c**) staining were positive, and Ki-67 (**d**) had a small amount of positive staining

The patient was a 51-year-old male who presented with an abdominal mass for 2 years and right upper abdominal pain for 1 month. Two years prior to this study, the abdominal ultrasonography of the patient showed an upper abdominal hypoechoic nodule, suggesting an enlarged lymph node. One and a half years prior to this study, the contrast-enhanced CT suggested that the liver and stomach space contained an irregular mass, suggesting lymphoma. One month prior to this study, the patient had pain in the right hypochondrial region with no obvious causes; the pain was intermittent and radiated to the umbilicus, with self-remission. The mass appeared in the right upper quadrant, and increased in size rapidly. After physical examination, one-7 cm mass could be felt in the right upper quadrant, and the texture was medium. In laboratory tests, there was elevated blood glucose antigen 125, 316.9 U/mL (reference value, 0.0 – 35.0 U/mL). The routine abdominal CT and contrast-enhanced CT showed that a lobulated soft tissue mass was in the liver and stomach space with a size of approximately 7.7 × 14.2 cm^2^; the internal density was slightly heterogeneous, and the boundary was still clear. In CT arterial phase, the peripheral part of the mass had slight reinforcement. Gross vascular features were seen in the center of the lesion. During the portal phase and delay phase, there was progressive enhancement of lesions and reinforcement came from the center to the surrounding progress, showing a centrifugal reinforcement (Fig. 1). Other manifestations included chronic liver disease, splenomegaly, ascites, a cavernous transformation of the portal vein, and a collateral circulation opening. CTA showed that the vascular features were in the center of the lesion, which had blood supplied by the anterior superior pancreaticoduodenal artery and the left gastric artery (Fig. 2). An abdominal MRI scan showed a huge lobulated mass in the liver and stomach space compared to the muscle signal; the T1-weighted imaging (T1WI) signal was isointense, T2-weighted imaging (T2WI) was slightly hyperintense, and there were clear boundaries. A diffusion-weighted imaging (DWI) sequence of the mass showed slight hyperintensity. In a contrast-enhanced MRI, as time passed, the lesion was enhanced from the center to the periphery, and the degree of enhancement was enhanced, showing centrifugal enhancement (Fig. 3). After completing the necessary examinations and discussing operation options with the patient, excision of the abdominal mass was carried out. During the operation, there was a small amount of ascites, and there were no obvious tumor nodules in the peritoneum, mesenteric and intestinal wall. The tumor was located between the liver and stomach; it was lobulated, hard, and closely adhered to small curved side of the stomach. Gross observation showed a mass with a size of 5.5x9.5x15 cm^3^, and the cutting section was gray. Pathological magnification showed the tumor cells were spindle. Cytoplasmic eosinophilic red showed there was substantial differentiation, but nuclear division was rare. The interstitial vascular, spindle cells and interstitial blood vessels were mixed in different proportions, and the regional blood vessels formed a dense vascular-network-like structure with some spindle cells around blood vessels protruding into the lumen of blood vessel arrangement. The spindle cells were abundant in some areas, showing bundled or braided arrangements. The immunohistochemical results were, smooth muscle actin (+) desmin (+), Ki-67 (+, few), S-100 protein (−), CD34 (−), CK (−), progesterone receptor (−), estrogen receptor (−), MelanA (−), HMB45 (−), CD117 (−), and DOG-1 (−) (Fig. 4). The final pathological diagnosis was myopericytoma. The patient was followed up with for 5 months, and there was no recurrence or metastasis during that period.

## Discussion and conclusions

At present, the etiology of myopericytoma is unknown, and there are some cases that have reported that it may be related to trauma and virus infection [9, 10]. Myopericytoma is a rare tumor, which can occur at all ages, and is more common in middle-aged men [4]. Myopericytomas are mostly seen in the distal lower extremities, located in the skin and dermis, and rarely in deeper locations [4]. The clinical manifestation of a myopericytoma involves the slow growth of painless nodules, and some patients may have pain or tenderness [11]. The occurrence of myopericytomas in adults is usually as a solitary tumor; if occurring in the children, myopericytoma occasionally shows multiple lesions [11]. In this case, the age of the middle-aged male patient was the same as the reported age of onset. However, in this case, the myopericytoma occurred in the abdominal cavity, and no similar reports were found. Similar to previous reports, this case had no clinical manifestation for a long time, but in the later stages of tumor development, the mass significantly increased, and symptoms of abdominal pain appeared. In this case, the development of the tumor suggested that deep myopericytoma was not recommended for clinical observation. The tumor might have accelerated growth in the late stages of the disease, so the tumor needs to be removed early. Myopericytomas were mostly benign tumors, and the typical morphological pathological characteristics included many thin-walled vessels, an ovoid shape, and spindle tumor cells with concentric or swirling growth around the peripheral blood vessel [4]. For immunohistochemistry, the tumors were usually positive for ASMA and h-caldesmon staining. The results of typical morphology and immunohistochemistry can prompt the diagnosis of myopericytoma, but the diagnosis needs to be differentiated from myofibroma and angioleiomyoma [4].

The image findings of myopericytoma have been rarely reported, and the case studies that did report imaging findings had tumors distributed in different locations of the body [5, 12–16], including 7 cases in the head and neck, 6 cases in the chest, 3 cases in the waist, and 2 cases in the lower extremities. Lesions can manifest as solitary round, oval, or irregular shapes, and occasionally there were multiple lesions [5, 12–16]. The size was 0.5 × 0.5–4.1 × 5.3 cm^2^ [5, 12–16]. Ultrasound examination showed the lesions were usually homogeneous hypoechoic with clear boundaries [17–19] and with obvious blood flow [17, 18]. Conventional CT examinations found that the lesions were usually isodense or hypodense, homogeneous or heterogeneous, occasionally calcified, and clearly bounded [14, 18, 20–24]. In contrast-enhanced CT scans, lesions were shown as homogeneous with severe enhancement [5, 17–20] or peripheral enhancement [5, 14, 15, 22]. Small vessels may be seen around the lesion [20]. In the reports, small lesions usually showed significant enhancement overall, while some large lesions showed peripheral enhancement due to central ischemic necrosis. In conventional MRI, T1WI, lesions usually showed hypointensity or slight hypointensity, T2WI had hyperintensity, and the signal was heterogeneous [5, 13, 16, 25]. On contrast-enhanced MRI, the lesions showed severe enhancement [5, 13, 21]. When the vertebral body was involved, there was an osteolytic lesion and it could be compressed to the spinal cord [12, 16, 25, 26]. Lesions occasionally had hemorrhage [13] or lymph node enlargement [15]. PET examinations showed that lesions usually showed moderate fluorodeoxyglucose (FDG) uptake [21, 24]. In the current case, lesions in the contrast-enhanced CT and MRI were both rich in blood supply, and clear blood vessels were seen. However, the enhancement pattern of this case showed there was centrifugal enhancement, and no similar reports were found in the literature. In previous case studies, it was believed that the centrifugal enhancement was the characteristic enhancement pattern of focal nodular hyperplasia (FNH) in liver diseases. To the best of our knowledge, this study is the first time that centrifugal enhancement has been reported for a myopericytoma. However, further evaluation of the diagnostic value of centrifugal enhancement for deep myopericytomas is needed. Preoperative imaging of myopericytomas should be distinguished from other tumors with rich blood supplies.

Surgical resection of myopericytomas has a good curative effect, and occasionally there is recurrence or rarely metastasis [4]. In this case, a deep myopericytoma in the abdominal cavity was found and there were no obvious signs of malignancy. However, in the late stage of the case tumor size increased significantly, and therefore, although most of the tumor was benign, early resection was still recommended. Another study found that a BRAF^WT/V600E^-myopericytoma should be closely followed up with because of the risk of recurrence [27].

The image findings for myopericytoma usually show a rich vascular lesion, and when the tumor center was necrotic, there is typically peripheral enhancement. A myopericytoma of the abdominal cavity could show centrifugal enhancement. Early resection of the tumor was necessary because it may have increased rapidly in the later stages of the disease.

## Abbreviations

CT: Computed tomography
CTA: Computed tomography angiography
DWI: Diffusion-weighted imaging
FDG: Fluorodeoxyglucose
FNH: Focal nodular hyperplasia
MRI: Magnetic resonance imaging
T1WI: T1-weighted imaging
T2WI: T2-weighted imaging
